# Water Turbidity and Suspended Particulate Matter Concentration at Dredged Material Dumping Sites in the Southern Baltic

**DOI:** 10.3390/s22208049

**Published:** 2022-10-21

**Authors:** Barbara Lednicka, Maria Kubacka, Włodzimierz Freda, Kamila Haule, Grażyna Dembska, Katarzyna Galer-Tatarowicz, Grażyna Pazikowska-Sapota

**Affiliations:** 1 Department of Physics, Gdynia Maritime University, Ul. Morska 81-87, 81-225 Gdynia, Poland; 2 Department of Operational Oceanography, Maritime Institute, Gdynia Maritime University, Ul. Długi Targ 41/42, 80-830 Gdańsk, Poland; 3 Department of Environmental Protection, Maritime Institute, Gdynia Maritime University, Ul. Trzy Lipy 3, 80-172 Gdańsk, Poland

**Keywords:** Baltic Sea, turbidity, suspended particulate matter, nutrients, dredged material dumping

## Abstract

Dredged material dumping is an activity that causes some of the greatest changes in coastal waters. It results in the need to regularly monitor the properties of seawater related to water quality. In this study, we present the first wide-ranging attempt to correlate seawater turbidity and suspended particulate matter (SPM) concentrations within dumping sites and adjacent waters on the basis of in situ measurements. In the years 2019–2020, we examined four dumping sites, namely Darłowo, Gdynia, Gdańsk, and DCT, located in Polish coastal waters of the Baltic Sea, in the course of four measurement campaigns conducted in the spring, summer, autumn, and winter. The measurements were conducted using a turbidity sensor to determine the turbidity, in formazin turbidity units (FTU), a spectrophotometer to determine the concentrations of nutrients (total phosphorus (P-tot), phosphate phosphorus (P-PO_4_^−3^), total nitrogen (N-tot), ammonium nitrogen (N-NH_4_^+^), and nitrate nitrogen (N-NO_3_^−^)), as well as glass microfiber filters to determine the concentrations of SPM. The analysis of the relationship between the turbidity and SPM within the dumping sites in comparison to reference points showed that the dumping sites are very complex waters and that the issue must be approached locally. The highest turbidity values were registered in the spring, and they correlated linearly with the SPM concentrations (R^2^ = 0.69). Moreover, we performed a statistical cluster analysis to illustrate the similarities between sampling points in the four dumpsites based on nutrient concentrations. We conclude that the influence of the dumping sites on the local bio-optical and chemical properties significantly exceeds their borders and spreads to the adjacent waters. Nutrient concentrations in many cases exceeded the legal policy limits.

## 1. Introduction

Each year, a significant amount of dredged material is deposited on the seabed in specially designated areas called sea dumping sites. The main source of deposited sediments is material extracted from ports and navigation channels to keep them operational [1,2,3,4]. Such human activities may have a potentially negative impact on water quality and marine life; this is why they require specific attention and are subject to legal restrictions (e.g., HELCOM). The dumping of sludge at sea, among other actions, increases turbidity, the amount of suspended solids, and the deposition of fine sediment over the designated dumping sites [5,6].

An increase in turbidity causes a decrease in the transparency of water, which harms pelagic organisms such as phytoplankton, zooplankton, fish, and birds foraging in the water column [7]. In the areas where spoil is stored, it is recommended to monitor the turbidity and take actions to minimize its increase, which is caused by the disposal of dredged material at sea [8]. The optical properties of natural waters are strongly connected to the biogeochemical and biogenic composition of water [9,10,11], which can provide information on the concentrations and types of suspended and dissolved particulate matter. The biochemical and optical properties of coastal waters, where underwater dumpsites are located, also depend on the complexity of the composition and types of optically significant components, such as phytoplankton, organic detrital particles, inorganic particles, and colored dissolved organic matter [12]. SPM includes particles larger than 0.45 µm, which form the suspended phase in water [13]. SPM consists of organic particles (bacteria, small organisms or their fragments, and plant pollen) and inorganic particles (dusty and clay material, sand and rock particles, and colloidal particles of various chemical compounds) suspended in the water mass.

The Baltic Sea is struggling with a growing problem of eutrophication [14,15]. The coastal waters of the Southern Baltic and its river estuaries are areas of high diversity and dynamic changes in water physics and chemistry, providing challenging environments for the study of the optical properties of water [16,17,18]. These areas are characterized by specific physical conditions, such as limited penetration of light through the water column and enhanced transport, as well as sedimentation of light organic and inorganic particulate solids. Therefore, measuring turbidity in coastal waters is essential, as it provides information about the concentration of suspended sediments in the water.

Contaminants in sediments may be harmful to aquatic organisms of the first three trophic levels (phytoplankton, zooplankton, and fish). Moreover, they can be sources of chemicals for bioaccumulation in the food chain. Therefore, different phytobenthic and zoobenthic species have different sensitivities to different toxicants [19]. Most of them are exposed to contaminants by the direct or inadvertent ingestion of sediments, as well as by uptake from pore water and overlying water [20]. The susceptibility of phytobenthic and zoobenthic organisms to contaminants differs for different species because of their “lifestyles” and possible contaminant exposure pathways as well as contaminant concentrations [19].

The knowledge about many of the relevant aspects associated with sea dumping sites is limited. To the best of our knowledge, this work is the first one to undertake the monitoring of dumpsites in Polish coastal waters, except for the Gdynia dumpsite, which was investigated in 2011 as part of the ECODUMP project and in the Pomeranian Bay [3]. This causes gaps in reliable local theoretical models for predicting the influence of sea dumping sites on the optical properties of seawater. Therefore, it is essential to check whether and how the periodic injection of waste materials into the Baltic Sea impacts the coastal environment.

Because the variations in water turbidity clearly indicate certain changes in water constituents, it is important to examine how dredged material influences the optical properties of these complex waters. It is clear that this problem should be addressed locally after a regional analysis of the relevant parameters.

In our research, we examined the turbidity of four dumpsites located in Polish coastal waters, which vary in size, depth, and location. The study was carried out four times, once in each season. In this paper, we titled the campaigns after the names of the seasons: spring, summer, autumn, and winter. We conducted turbidity measurements in the entire water column and additionally collected water samples at two different depths (the surface and the seabed) for the analysis of SPM and the following nutrients: P-tot, P-PO_4_^−3^, N-tot, N-NH_4_^+^, and N-NO_3_^−^. Our tests and analyses were essentially focused on the use of optical and biochemical measurements in complex aquatic environments of sea dumping sites and waters in the Southern Baltic to develop new capabilities for monitoring the statuses of these areas, including their biogeochemical and water quality parameters.

This article answers the following questions: (a) What is the effect of dredging material dumping sites on water turbidity and water constituents? (e.g., SPM concentrations and concentrations of nutrients) (b) What are the local relationships between turbidity and seawater constituents, and (c) do they change temporary? The paper discusses the first such detailed study on turbidity in Polish coastal waters, with a focus on its temporal variability. The results presented in our paper make a unique contribution to the environmental assessment of the impact of dumpsites on the water quality of the coastal waters of Baltic Sea.

## 2. Materials and Methods

### 2.1. Dumping Sites

**Figure 1:**
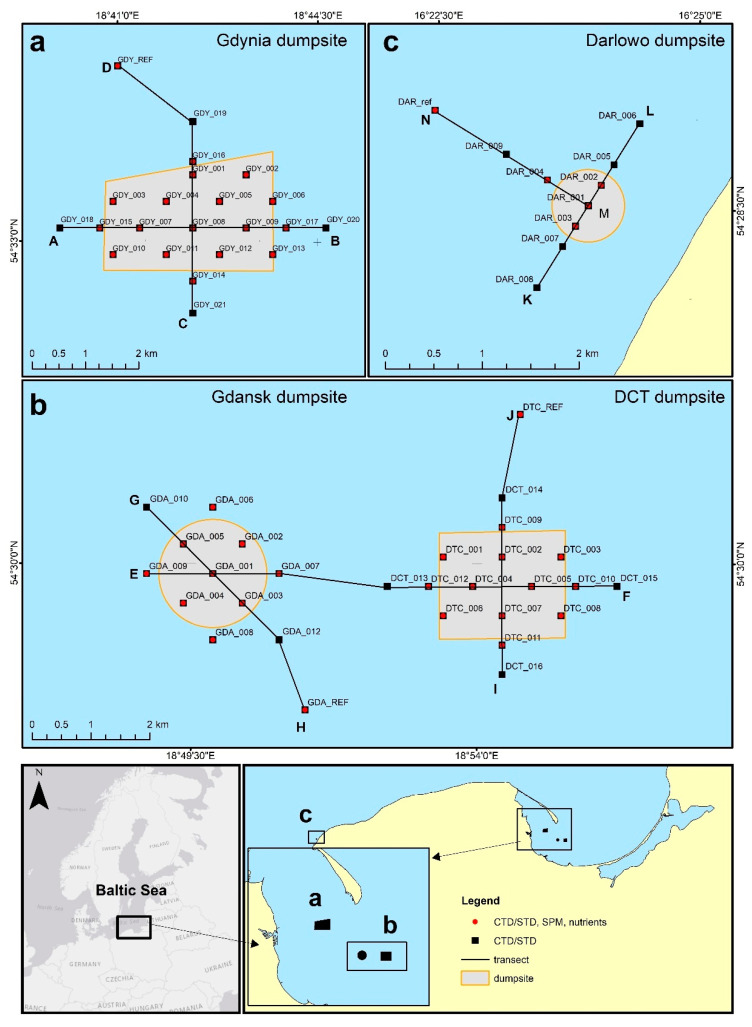
Map of the study sites, with the survey transects and the sampling points marked by red and black dots.

Empirical data were collected from aboard the IMOR r/v and IMOROS 2 motorboats from over 61 stations (Figure 1) during four campaigns: summer (22–29 August 2019), autumn (20 October–17 November 2019), winter (20–29 February 2020), and spring (26 April–22 May 2020). Four sea dumping sites in the Southern Baltic were surveyed, which varied in size, depth, and location (see Figure 1).

The smallest of them (0.43 km^2^) was the dumping site of Darłowo, which is located in the central part of the Polish coast of the Baltic Sea, approximately 4 km from the coastline (Figure 1c). It covers a fragment of the seabed at depths varying from approximately 7 m to 14 m. The average depth of the area is approximately 10.5 m; this makes it the shallowest of the surveyed test sites. Due to its location, the area is characterized by high water dynamics, which favor the mixing of water in vertical planes and exchange with waters of the Southern Baltic. In the time preceding our measurements, no material discharges were recorded at the Darłowo dumpsite.

The surveys also covered three dumping sites in the area of the Gdańsk Bay, i.e., the Gdańsk, Gdynia, and Deepwater Container Terminal (DCT) dumpsites. The largest one is the Gdynia dumping site (6.4 km^2^), which is the most western of the three dumping sites (Figure 1a). It covers a fragment of the seabed, spreading at a depth from approximately 27 m to 52 m. The average depth at which the survey stations are located is approximately 30 m. The northern side of the area is shielded by the Hel Peninsula. Dredged material was dumped at the Gdynia dumpsite 12 times from October to November 2019 and 18 times from April to May 2020. There is no information on any dredged material in August 2019 and February 2020. The last dumping of material in the autumn campaign occurred 3 days before the measurement, and for the spring campaign this was 2 days before. The Gdańsk dumping site (2.7 km^2^) is located in the southern part of the Gdańsk Bay (Figure 1b). The average depth of the survey area is approximately 31 m. Dredged material was dumped at the Gdańsk dumpsite 26 times in August 2019, 8 times from October to November 2019, and 6 times in February 2020; there were no instances from April to May 2020. The DCT dumping site (4.0 km^2^) is the deepest one, and it covers a fragment of the seabed at a depth from approximately 58 m to 65 m. It is the most eastern of the three test sites in this area (Figure 1b). Dredged material was dumped at the DCT dumpsite 102 times in August 2019, 252 times from October to November 2019, 56 times in February 2020, and 73 times from April to May 2020. This being the case, the frequency of discharge was a few times a day, such that it directly preceded the taking of the measurements.

### 2.2. Turbidity Measurements

Hydrophysical surveys were performed within the areas of the dumping sites and adjacent waters at points distributed at least 0.2 km from one another, as well as at reference points located 1.85 km from the area of the sea dumping site. They form two intersecting transects in each dumpsite (Figure 1, Table 1).

We measured the turbidity in the entire water column and collected water samples from the near-surface and near-seabed layers of the water in each of the survey campaigns. Turbidity measurements were conducted at 61 sampling stations, whereas the concentrations of total SPM and concentrations of nutrients of the subsurface and near-bottom layers were measured at 46 sampling points (including four reference points). The following statistical analysis has been performed for these 46 stations, containing complete turbidity and biogeochemical information.

Two sets of sensors were used during this research. The multiparameter SAIV SD204 CTD/STD probe (SAIV A/S, Bergen, Norway) [21] was used in the summer and autumn campaigns, while in the winter and spring campaigns, we used the Valeport MIDAS CTD Profiler probe (Valeport Ltd., Totnes, UK) [22]. The devices are equipped with a nephelometer with a sensor that measures the intensity of the light scattered by suspended solids in formazin turbidity units (FTU) [23]. We used it for the measurement of S, pressure, and turbidity (Midas in the range of 0–2000 FTU, SAIV in four selectable ranges: 12.5, 62.5, 250, and 750 FTU). Both of these sensor kits are equipped with turbidity sensors manufactured by Seapoint Sensors Inc. They measure turbidity by detecting the light scattered from suspended particles in water. The light source is an LED that emits light with a wavelength of 880 nm. The detectors are silicon photodiodes with visible-light-blocking filters. The detector can register light scattered at angles from 15 to 150 degrees. The amount of scattered light that reaches the detector is proportional to the turbidity of the water up to 750 FTU [21,22]. When the instrument was lowered to the bottom, the data were recorded every second. The sampling frequency was constant (six measurements per meter).

The collected hydrophysical data were interpolated using the kriging method in the Surfer program to obtain vertical cross-sections of the distribution of the turbidity in the entire water column along the designated transects.

### 2.3. Biogenic Measurements

Water samples were collected at the sampling points (red dots in Figure 1). The dry weight of the SPM was calculated by using the gravimetric method in compliance with the Polish Standard introducing the European Standard [24]. The concentration of the SPM was measured gravimetrically after the filtration of water through preweighed filters. Glass microfiber filters, GF/F (47 mm in diameter), prepared specifically for this purpose were used. Premeasured volumes of seawater were filtered immediately after sample collection. The concentrations of selected nutrients (P-tot, P-PO_4_^−3^, N-tot, N-NH_4_^+^, and N-NO_3_^−^) were analyzed only in the near-seabed water layers using the spectrophotometric method by Spectroquant Pharo 100 (MERCK, Darmstadt, Germany) according to the appropriate procedures described in [25].

## 3. Results

In this part of the paper, we describe the results of the turbidity measurements performed at all of the measuring stations of the four dumping sites. We then present the near-bottom layer nutrient concentration values and apply a statistical analysis. Our research focuses on the range of the temporal and spatial variability in the studied parameters.

### 3.1. Turbidity Depth Profiles

**Figure 2:**
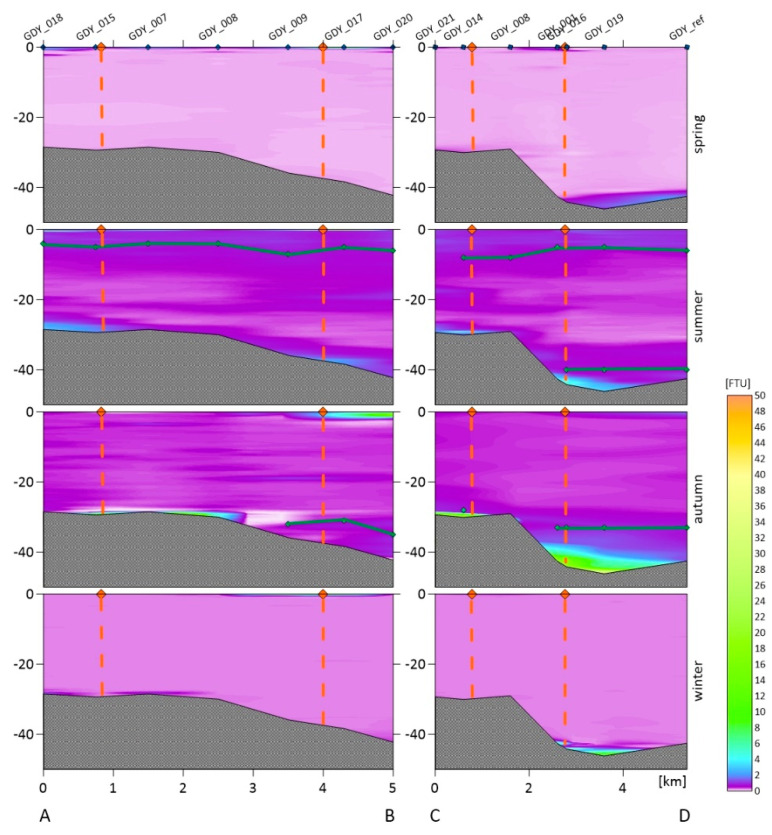
Vertical cross-sections of the turbidity of the seawater obtained based on the in situ measurements conducted along the AB and CD sections. The orange points at the sea surface and dashed lines are the borders of the Gdynia dumpsite, the green line is the halocline, and the measurement points along the transects are marked in blue on the top axis.

**Figure 3:**
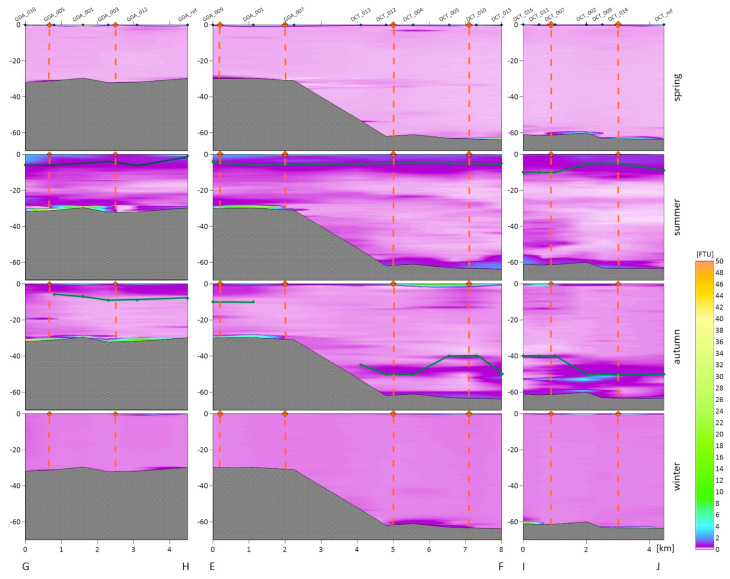
Vertical cross-sections of the turbidity of the seawater obtained based on the in situ measurements conducted along the GH, EF, and IJ sections. The orange points at the sea surface and dashed lines are the borders of the Gdańsk and DCT dumpsites, the green line is the halocline, and the measurement points along the transects are marked in blue on the top axis.

**Figure 4:**
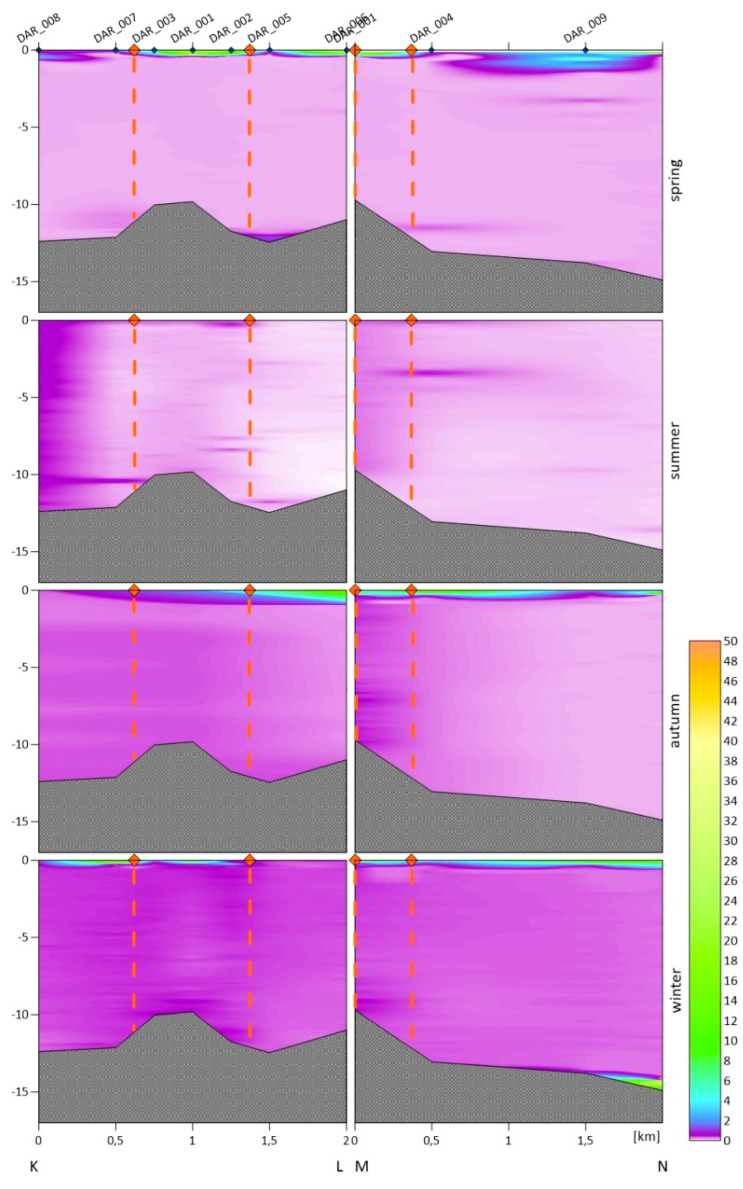
Vertical cross-sections of the turbidity of the seawater obtained based on the in situ measurements conducted along the KL and MN sections. The orange points at the sea surface and dashed lines are the borders of the Darłowo dumpsite; the measurement points along the transects are marked in blue on the top axis.

For each dumpsite, we obtained the vertical variability in the turbidity in the water column. The following figures show vertical cross-sections of the turbidity for two transects of the Gdynia dumping site (Figure 2), three transects of the Gdańsk and DCT dumping sites (Figure 3), and two transects of the Darłowo dumping site (Figure 4). The results are divided into measurement campaigns: spring, summer, autumn, and winter.

Haloclines only occurred in the summer and autumn in the dumpsites located in the Gdańsk Bay area. For an emerging halocline, its depth is marked in Figure 2 and Figure 3 as a green line. The vertical profiles show that higher values of turbidity are observed near the seabed and the surface, and sometimes near the halocline. In the winter and spring, we did not observe as high differentiation as in the summer and autumn in the entire cross-section of the water body. We recorded higher turbidity values both in the area of dumping dredged material and beyond its borders.

In the area of the Gdynia dumpsite (see Figure 2), the highest value of turbidity averaged over all of the depths and all of the measurement points within the dumpsite was recorded in the autumn (1.59 FTU). The highest locally measured turbidity, of 34.7 FTU was also observed in the autumn. High values were also observed in the summer just outside the borders of the dumpsite. High variability in the turbidity was especially observed near the surface and in the bottom layers. A smaller differentiation in the entire water column occurred in the spring and winter. The lowest value of the turbidity averaged over all of the depths and all of the measurement points within the dumpsite was recorded in the winter (0.55 FTU).

Turbidity cross-sections for both the DCT and Gdańsk dumping sites are presented in one long transect, i.e., EF, oriented from W to E, and two shorter transects analyzed separately for both of the dumpsites: GH (Gdańsk) and IJ (DCT) (Figure 3). The highest local value of the turbidity, of 16.7 FTU, was recorded in the near-bottom layer in the summer campaign. Similar to the Gdynia dumping site, large variability in turbidity in the vertical profile in the summer and autumn campaigns is also observed here.

The highest value of the turbidity at the Gdańsk dumpsite averaged over all of the depths and all of the measurement points within the dumpsite was recorded during the summer campaign (1.40 FTU). The mean turbidity value calculated in the same way for the DCT dumpsite, which is approximately 30 m deeper, was 0.38 FTU in that campaign. For both of the dumpsites, the turbidity distribution in the water column was similar; higher values were observed in the near-seabed and near-surface layers. A smaller differentiation in the entire water column was observed in the spring and winter. At the DCT dumpsite, the average turbidity values were similar throughout the year, with a peak in the summer (0.38 FTU) and a fall in the spring (0.25 FTU). Much greater differences were observed at the Gdańsk dumpsite, where the highest mean values were recorded in the summer (1.40 FTU) and the lowest in the winter (0.26 FTU).

In Figure 4, two cross-sections are presented for the Darłowo dumpsite: the KL section, orientated from SW to NE, parallel to the shoreline, and the MN section, orientated from SE to NW, perpendicular to the shoreline. Both transects are 2 km in length. At the Darłowo dumpsite, the highest value of the turbidity averaged over all of the depths and all of the measurement points within the dumpsite was recorded during the winter campaign (0.42 FTU), and the lowest during the summer and spring surveys (0.25 FTU). The recorded average turbidity values were similar across the water column in all four of the campaigns (~0.34 FTU). However, similar values averaged over the entire water column do not reflect the variability in the profile. High values were observed at the water surface in the spring, autumn, and winter, while the waters below that layer were characterized by low turbidity with little variation in the profile. Especially low values of turbidity are observed there in the spring and summer. In the MN transect, lower turbidity values were observed with increasing distance from the shore. No halocline was noticed in any survey campaign.

### 3.2. Concentrations of Nutrients

**Figure 5:**
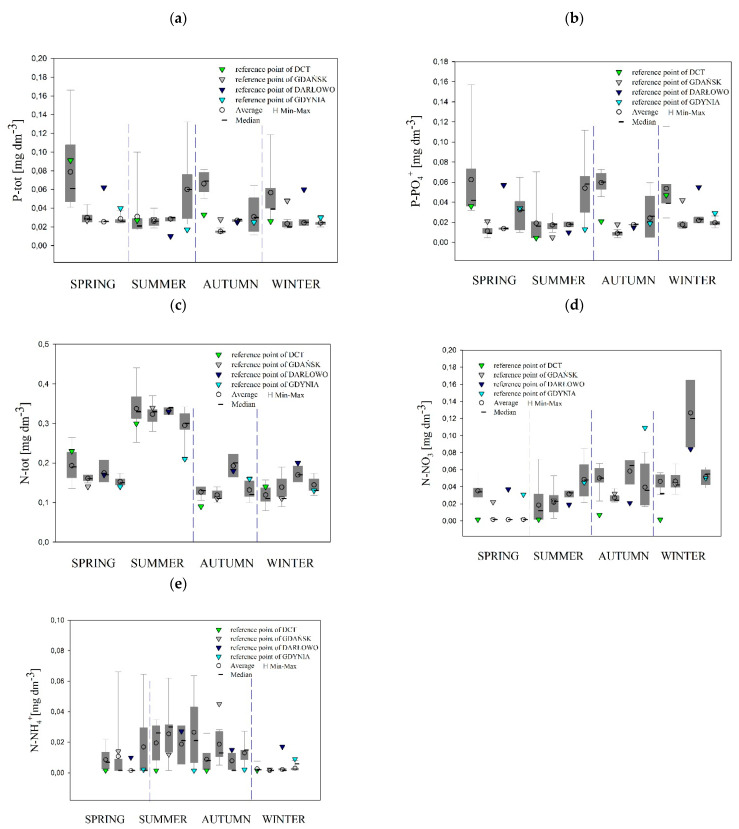
Concentrations of nutrients (**a**) P-tot, (**b**) P-PO_4_^−3^, (**c**) N-tot, (**d**) N-NO_3_^−^, and (**e**) N-NH_4_^+^ in the collected samples of the four dumping sites. The results are divided into seasons, and for each season of the year the graphs, starting from the left, represent DCT, Gdańsk, Darłowo, and Gdynia, respectively. Vertical bars depict the variation ranges, circles show average values, and dashes depict medians of concentration. The vertical boundaries of the rectangles show the range of 25–75% of concentration. The triangles represent the concentration value measured at reference points outside of the dumping sites.

All of the statistical analyses were performed for 46 stations (including four reference points), containing complete turbidity and biogeochemical information. During the cruises, the concentrations of biogenic substances were measured in the near-bottom layer of the seawater. In Figure 5, we show the range of the variability, average values, and median of nutrients P-tot, P-PO_4_^−3^, N-tot, N-NO_3_^−^, and N-NH_4_^+^ in the collected samples of the four dumping sites, divided into seasons. The highest temporal and spatial variability in P-tot, P-PO_4_^−3^, and N-NO_3_^−^ was observed. However, the nutrient concentrations were not significantly different among the dumpsites (Figure 5a,b,d). The largest differences between the median and average concentrations of all of the nutrients were observed in the spring, especially in the DCT dumpsite, when the median and average concentrations, e.g., for P-tot, were 0.061 mg·dm^−3^ and 0.079 mg·dm^−3^, respectively.

Table 2 lists the concentrations of nutrients that come from the bottom layers only. These concentrations are averaged for all of the measurement points belonging to the given dumping sites. The average values have been compared with the values measured at the reference stations (after the slash).

The temporal variations in nutrient concentrations and light determine the annual life cycle of phytoplankton, which is similar in the entire Baltic Sea. It is characterized by an intensive short peak bloom of diatoms in the spring (caused by adequate light and adequate concentrations of nutrients), after which other algal blooms occur from the mid-summer to the autumn. Following the spring bloom, the concentrations of nutrients in the water decrease sharply. Their levels remain low till the autumn due to insignificant vertical water motion in the summer. In the autumn, due to the increased mixing of water masses, regenerated nutrients rise from the near-seabed layers and enrich the euphotic zone of the sea. Their income is large enough that the autumn production, which is less intensive than the spring bloom, does not exhaust the reserves of nutrients. The winter inhibition in primary production (caused by much less light and much lower temperatures) enables the full regeneration of nutrient reserves in the euphotic zone [26,27].

In the dumpsites of Darłowo and Gdynia, the concentrations of nutrients in the tested water indicate temporal variability, which is specific to the Southern Baltic. The lowest concentrations of the tested substances occurred in the summer, while in the autumn and winter a significant increase was observed in accordance with the seasonal trend of the restoration of nutrients. In the Gdańsk dumping site, the concentrations of nutrients in the tested water indicated temporal variability, but with certain disturbances. The lowest concentrations of P-PO_4_^−3^ occurred in the autumn, while an increase in its concentration was observed in both the winter and summer. At that dumping site, the concentrations of nutrients in the water analyzed did not show temporal variability, typical for the waters of the Southern Baltic. The average concentrations of P-PO_4_^−3^ and P-tot in the near-seabed waters of the DCT site were similar throughout the entire period. In the case of N-NH_4_^+^ and N-tot, increases in concentrations were recorded in the summer, probably as a result of an increase in the amount of organic matter after the spring phytoplankton bloom.

The mean values of N-tot are comparable with those of the data in the literature. The Chief Inspectorate of Environmental Protection [28] provides the concentration of N-tot in the surface waters of the Outer Puck Bay in the summer of 2018, 0.66 mg∙dm^−3^. Zalewska and Kraśniewski [29] report the concentration of N-tot, 24.56 µmol∙dm^−3^ (0.34 mg∙dm^−3^), in the surface waters of the Gdańsk Basin in the summer, and 27.71 µmol∙dm^−3^ (0.39 mg∙dm^−3^) as the average for the decade of 2008–2017 for this water body. Krzymiński [30] provides the concentration of N-tot in the waters of the Bornholm Basin in the summer of 2016 as 25.91 µmol∙dm^−3^ (0.36 mg∙dm^−3^). Similar values of N-tot were found in the waters of the Arkona Basin, with 20.4 μmol∙dm^−3^ (0.29 mg∙dm^−3^), and in the Bay of Mecklenburg, with 29.4 μmol∙dm^−3^ (0.41 mg∙dm^−3^) [31].

The presence of N-NO_3_^−^ in the tested samples was found only in the winter, when the nutrient pool was being rebuilt. The mean value of the concentration of N-NO_3_^−^, both in the areas of the dumping sites and at the reference points, was similar to the concentration of N-NO_3_^−^ (approx. 0.006 mg∙dm^−3^) given in the waters of the Arkona Basin in the southwestern Baltic by Gustafsson [32]. Lower values of N-NO_3_^−^ are provided in the report of the Chief Inspectorate of Environmental Protection [28] in the waters of the Outer Puck Bay in the winter of 2018: 0.002 mg∙dm^−3^.

The concentrations of P-PO_4_^−3^ are reported by Zalewska and Kraśniewski [29] at the level of 1 µmol∙dm^−3^ (0.094 mg∙dm^−3^) in the surface waters of the Gdańsk Basin in winter and 0.57 µmol∙dm^−3^ (0.054 mg∙dm^−3^) as the average for the decade of 2008–2017 for this body of water. Krzymiński [30] gives the average value of P-PO_4_^−3^ in the waters of the Bornholm Basin for the winter months of 2006–2015: 0.42 µmol∙dm^−3^ (0.04 mg∙dm^−3^). Wasmund et al. [33] report the concentration of P-PO_4_^−3^ in the waters of the Arkona Basin in January–February: 0.64 µmol∙dm^−3^ (0.061 mg∙dm^−3^). However, in May 2016, the content of P-PO_4_^−3^ in the waters of the Arkona Basin was 0.29 µmol∙dm^−3^ (0.028 mg∙dm^−3^) in the surface layer and 0.48 µmol∙dm^−3^ (0.046 mg∙dm^−3^) in the bottom layer, and in the Bornholm Deep it was 0.40 µmol∙dm^−3^ (0.038 mg∙dm^−3^) in the surface layer and 2.28 µmol∙dm^−3^ (0.22 mg∙dm^−3^) near the bottom [34].

The obtained results of the concentration of P-tot are comparable with the data in the literature. The Chief Inspectorate of Environmental Protection [28] provides the concentration of P-tot in the surface waters of the Outer Puck Bay in the summer of 2018: 0.046 mg∙dm^−3^. Zalewska and Kraśniewski [29] report the concentration of P-tot as 1.17 µmol∙dm^−3^ (0.036 mg∙dm^−3^) in surface waters of the Gdańsk Basin in the summer and 0.80 µmol∙dm^−3^ (0.025 mg∙dm^−3^) as the average for the decade of 2008–2017 for this body of water. Krzymiński [30] gives the average concentration of P-tot in the summer period in the waters of the Bornholm Basin in the years 2006–2015 as 0.85 µmol∙dm^−3^ (0.026 mg∙dm^−3^). Similar values of P-tot concentrations were found in summer in the waters of the Arkona Sea: 0.7 µmol∙dm^−3^ (0.21 mg∙dm^−3^) [35].

### 3.3. Turbidity in the Entire Water Columns and Tested Layers

The measurements of sea water turbidity, which were presented in the previous section, and the SPM concentration were averaged for one meter of the near-surface layers and for one meter of the near-bottom layers. Averaging was performed for all of the sampling stations in the dumping site areas. For turbidity, the depth profiles shown in Figure 2, Figure 3 and Figure 4 were also averaged for the entire water column. All of these averaged values are summarized in Table 3. Such average values are compared in this table with the values measured at the reference points (after the slash). The turbidity values determined by us are similar to the turbidity ranges observed in the coastal waters of Sweden (between 0.3 and 27.8 FTU) and Lithuania (between 0.6 and 49.8 FTU) [36].

The highest values of turbidity, averaged over the entire water column and all of the measurement points inside a dumping site, were recorded at the dumpsite in Gdynia in the autumn (1.59 FTU) and in Gdańsk in the summer (1.40 FTU) as well as autumn (1.09 FTU). The lowest averaged values of 0.25 FTU were recorded at the Darłowo dumpsite in the summer and at the DCT dumpsite in the spring. Other very low values of turbidity were recorded at the Gdańsk dumpsite in the winter (0.26 FTU) and the spring (0.28 FTU). The greatest variation in the means throughout the year was observed in the Gdańsk dumpsite. In the remaining dumpsites of the Gdańsk Bay, these values are more similar throughout the year, such that the lowest averages were recorded in the spring and winter, and the highest in the summer and autumn. During the winter, a noticeable decrease in offshore turbidity was observed due to a reduction in the production of phytoplankton, which represent the main source of suspended particles that determine the variations in turbidity in areas far from the shoreline [37,38]. The opposite situation was observed in the Darłowo dumping site: in the summer and autumn we recorded the lowest mean values, and in the winter and spring we recorded the highest ones. A similar trend in the coastal zone was reported by Constantin et al. [39]. However, the turbidity there was influenced mostly by river discharges.

In all three of the dumpsites located in the Gdańsk Bay in the spring and winter seasons, the distribution of turbidity in the entire water column was similar. A higher level of turbidity in the near-seabed water layers was observed at selected stations, which may be related to the types of sediments in this location. Significant differences in the turbidity distribution in the water column occur in the summer and autumn seasons. We recorded higher turbidity values especially in places where the halocline forms [40]. A different situation was observed in Darłowo, where the vertical distribution of turbidity in the water column is similar throughout the year, and the turbidity decreases with the distance from the shore.

The tested near-surface layer is characterized by high values of turbidity for all of the dredged material dumping sites. The highest level of turbidity of 5.35 FTU in the top layer was recorded in the dumping site of Darłowo in the autumn (see Table 3). However, other high values in that layer are recorded at the dumping sites of Darłowo in the spring (3.85 FTU), Gdynia in the spring (3.74 FTU) as well as autumn (3.37 FTU), DCT in the autumn (3.65 FTU), and Gdańsk in the spring (3.53 FTU). The lowest levels were recorded in the near-surface layer in the dumping sites of Gdańsk in the winter (0.30 FTU) and Darłowo in the summer (0.44 FTU). The increase in the turbidity in the surface layers is probably influenced by plankton, which accumulate above the halocline.

In the near-bottom layer, the highest level of turbidity was recorded in the dumping site of Gdańsk in the summer (8.66 FTU). However, very high turbidity levels (>3 FTU) were observed for the autumn in all of the dumping sites located in the Gdańsk Bay. This is due to a documented discharge of dredged material that took place on the day on which the measurements were taken. The lowest mean levels of turbidity in the near-seabed layer were recorded in the Darłowo dumpsite in the summer, spring, and autumn (<0.6 FTU).

### 3.4. Relationship between Turbidity and the SPM

**Figure 6:**
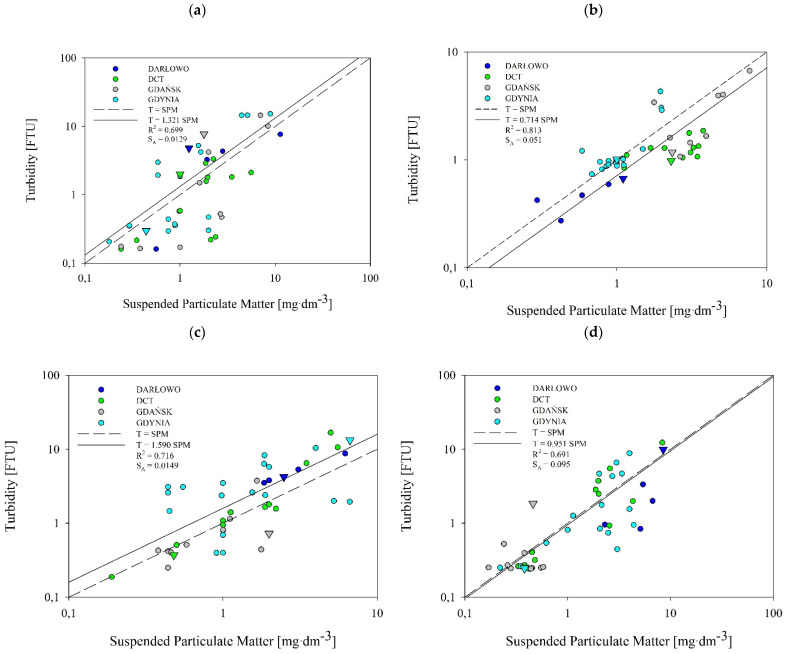
Correlations between in situ turbidity and the concentration of SPM in the near-surface layer for the spring (**a**), summer (**b**), autumn (**c**), and winter (**d**). Circles depict the sea dumping site points, while triangles depict reference points. Throughout this figure, T also means turbidity.

**Figure 7:**
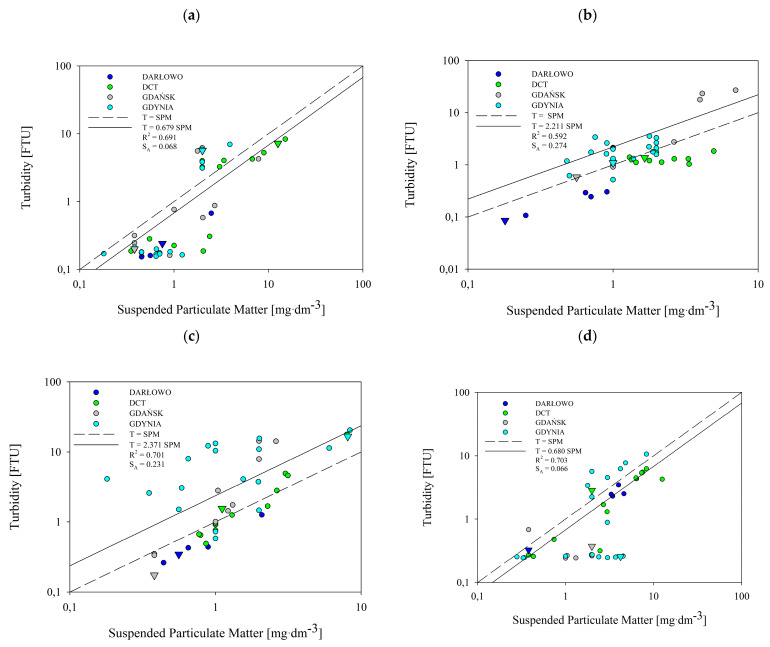
Correlations between in situ turbidity and the concentration of SPM in the near-seabed layer for the spring (**a**), summer (**b**), autumn (**c**), and winter (**d**). Circles depict the sea dumping site points, while triangles depict reference points. Throughout this figure, T also means turbidity.

Figure 6 and Figure 7 present the relationship between turbidity, T, and SPM concentration based on the data collected in all of the seasons and from all of the dumping site points (marked with circles) and reference points (marked with triangles). Using statistical analyses, we established a relationship between the T and SPM in four selected areas of the Baltic Sea. Turbidity tended to rise with increasing SPM. The spread of the measurement points is larger in the case of the near-seabed layer than the near-surface layer. The SPM in the water samples collected in all of the seasons ranged from 0.18 to 11.30 mg·dm^−3^ in the near-surface layer, and from 0.18 to 15 mg·dm^−3^ in the near-seabed layer. Turbidity ranged between 0.16 and 16.72 FTU (Figure 6) in the near-surface, and between 0.09 and 26.84 in the near-seabed layer (Figure 7). This range of variability is consistent with what is reported by Kari et al. [36] for Swedish and Lithuanian coastal waters of the Baltic Sea.

The majority of the reference points do not lay significantly apart on the charts from the rest of the points from the dumping sites. There are only two exceptions, namely in the near-seabed summer and autumn. In the summer, the reference point of the Darłowo dumpsite, along with a single point within this dumpsite, were clearly isolated from the other points (see the navy blue triangle in Figure 7b). The same was observed in the case of the Gdynia reference point in the autumn (see the grey triangle in Figure 7c). In the near-seabed layer in all of the seasons, most of the reference points were located below or on the regression line.

The analysis shows that the concentration of SPM correlates well with turbidity levels in the near-surface (Figure 6) and near-seabed (Figure 7) water layers. This was the case for all of the seasons. As shown in these figures, the correlation obtained has the form of a one-parameter linear function:

(1) T=A⋅SPM,

where T means turbidity, in FTU, and SPM is the concentration of suspended particulate matter, in mg·dm^−3^. The slopes, A, which were obtained by the least squares method, are presented with the correlation coefficients, R^2^, and standard errors of estimates, S_A_, in Figure 6 and Figure 7. The slopes, A, vary between 0.679 and 2.371. This is consistent with the results of Kari et al. (2017) [36]. However, they presented their linear relationship in logarithmic form. A similar linear relationship between the measured turbidity and SPM is given by Jafar-Sivik et al. (2017) [41]. They, in turn, present the SPM concentration as a function of turbidity, such that their slope coefficients are the inverse of our A coefficients.

The correlation coefficients, R^2^, between turbidity and SPM concentrations, calculated from 46 pairs of data for each season and each layer, range between 0.59 and 0.81. The highest correlation coefficient was observed in the summer for the near-surface layer (Figure 6b), and the lowest was observed in the summer for the near-seabed layer (Figure 7b).

The standard error estimate, S_A_, conforms to the values of the correlation coefficient, R^2^. For most of the results, the correlation coefficient is approximately 0.7. For these cases, S_A_ is approximately 10% of the slope, A. The exceptions are the two results obtained in the summer. For the subsurface layer in the summer season, the correlation coefficient is significantly higher and amounts to 0.813, while the standard error estimate is clearly lower than 0.1 A. Additionally, for the bottom layer in the summer season, the correlation coefficient is below 0.6. A low correlation value is accompanied by a higher scatter of points. Therefore, S_A_ exceeds 0.12 of the slope, A.

In the tested seawater samples taken from the areas of the four dumping sites and the reference points, generally low SPM concentrations were registered, in many cases being below 2.0 mg∙dm^−3^. This is especially visible in the near-surface water (see Table 3). These values are typical for the waters of the Southern Baltic region. On the contrary, the near-seabed layer was characterized by slightly higher levels of suspension, which may be related to the prevailing weather conditions, causing sediments to rise from the seabed. From this dataset of sea dumping sites of the Southern Baltic, we have observed a temporal variation in the relationship between SPM and turbidity. The dumping sites of Gdynia, Gdańsk, and DCT exhibit similar tendencies. The difference between them and the fourth dumping site can be explained by the fact that Darłowo is the shallowest of the surveyed sites. The dumpsites of Gdynia, Gdańsk, and DCT are sheltered by the Hel Peninsula, while the Darłowo dumpsite is characterized by high water dynamics, which favor the mixing of water in vertical planes and exchange with waters of the Southern Baltic.

## 4. Discussion

The aim of our research is to investigate the potential impacts of four dumping sites located in Polish coastal waters on the marine environment. For this purpose, we conducted survey campaigns in four seasons: the spring, summer, autumn, and winter. We collected relevant data at the measurement points set at and in the vicinity of each dredged material dumping site, as well as at reference points. We analyzed turbidity and the concentrations of SPM as well as nutrients. The research was conducted in such a way that allowed us to present the temporal distribution of turbidity throughout the water column and determine the local relationship between turbidity and SPM concentration.

### 4.1. Turbidity and SPM Concentration at the Dumpsites and Reference Points

Before starting the measurements, the places that were to be reference points were selected. These were to be places where, due to a slight distance (a distance of 1.85 km was established), turbidity and suspended solids should have values undisturbed by the presence of dredging material. After the measurements were taken, it was surprisingly found that both the turbidity and the SPM concentration measured for both the bottom and subsurface layers at reference points often exceeded the values measured within the discharge sites. A summary of the percentage number of measurement sites where the turbidity and SPM values exceed the respective reference point values is presented in Table 4. The values are given separately for the surface layer and bottom layer, with a distinction between the seasons and the sea dumping sites.

At the Gdynia and DCT dumpsites, during as many as three measurement campaigns, higher mean turbidity values were recorded in the water column than at the reference point, i.e., at the Gdynia dumpsite in the following seasons: the winter, spring, and summer, as well as at the DCT dumpsite in the winter, summer, and autumn. At the Gdańsk dumpsite, the mean exceeded the values recorded at the reference point in the autumn and summer, and the same was true at the Darłowo dumpsite in the autumn. The differences in the turbidity distribution within dumpsites and beyond their borders were most evident in Figure 2, Figure 3 and Figure 4. It is not a rule that within the dumpsites we observe a higher level of turbidity than in adjacent waters or at the reference points (see Table 4). This may indicate the spread of the deployed sediments beyond the boundaries of the dumpsites or the dumping of spoil outside the designated areas.

This leads to the hypothesis that the results of turbidity and SPM at the reference points do not differ statistically from the values measured in the areas of the dumping sites. In order to verify such a hypothesis, we first performed a Shapiro–Wilk test to verify that the data had a normal distribution. The null hypothesis, H_0_, and the alternative hypothesis were as follows. H_0_: the distribution of the examined feature is a normal distribution. H_1_: the distribution is not a normal distribution. For example, when analyzing the DCT site in the spring, we obtained n = 12 measurement points. The critical area in this test was the left-hand area, so we reject the null hypothesis if the calculated value of the statistic test is lower than or equal to the critical value, i.e., W ≤ W (α, n). In all cases, the confidence level, α, was equal to 0.05. The determined values of the W test statistic were 0.88 for SPM and 0.87 for turbidity (see Table 5). The critical value of W (α, n) was 0.859. The W values were higher than the critical value; therefore, there was no reason to reject the null hypothesis. In both cases, the measurement results were described by a normal distribution. This being the case, a *t*-test can be used to compare the results with a reference value. All of the W values and critical values of the test were collected in Table 5 and listed in black.

The Shapiro–Wilk test is considered to be the best test for checking the normality of the distribution of a random variable. The main advantage of this test is its high power, i.e., for a fixed α, the probability of rejecting the H_0_, if it is false, is higher than in the case of other tests of this type [42].

For measurements that have a normal distribution, the comparison of the reference value with the measurement results can be performed by using the *t*-test. For each of the cases for which the distribution of the obtained results is normal, the *t*-test results were compared with the critical value of the *t*-test distribution (see Table 5). The critical values of the distribution were determined on the basis of the confidence level of 0.05 and the number of degrees of freedom (n − 1). It turns out that statistically significant differences between the measurement results and the values measured at the reference point occur only for the Darłowo dumping site; they occur only in the winter and concern the bottom layer as well as turbidity in the surface layer (they do not apply to the SPM in the surface layer). These differences are shown in last raw of Table 5.

The *t*-test results clearly show that the predetermined positions of the points that were considered good reference sites were mistakes. Statistically significant differences only appeared in the Darłowo dumping site in the winter. This difference is visible in Figure 6d and Figure 7d. While in Figure 7d for the bottom layer, the turbidity and SPM values at the reference point are lower than those at the other points, it is the other way around in the near-surface layer (see Figure 6). Here, surprisingly, the values measured at the reference point are larger than those measured at other measurement sites. Despite the existence of statistical differences, the choice of reference site is also not appropriate here.

In future measurements, more measurements should be made outside the areas of spoil discharge sites, so that during the laboratory measurements and subsequent analyses of the results, conditions of sea currents, and wave directions, it is necessary to decide which point will be our reference.

Moreover, we applied the Kruskal–Wallis test to check the statistical differences between the measurements that were made on a given measurement point of the dumping sites and a given water layer, but at different times of the year. The Kruskal–Wallis test is used to compare three or more samples of results, and the null hypothesis is that all of the samples are from a population with the same median. The interpretation of this test is based on the ranks of the individual scores. In this way, we wanted to check whether there are statistical temporal differences in the turbidity and suspension of a given location. The null hypothesis is that there is no difference between four categories of the independent variable of measurement point in terms of two dependent variables of turbidity and SPM concentrations. The alternative hypothesis is there is a difference between the four categories of the independent measurement points in terms of two dependent variables of turbidity and SPM. We took a confidence level of 0.05, the same as in the Kruskal–Wallis test and *t*-test. In all cases, we compared the four categories with each other. Therefore, the critical value that comes from the χ^2^ distribution was the same in all cases and amounted to 7.815. For a value of H lower than the critical value, the null hypothesis cannot be rejected (Table 6).

The conclusion from this test is as follows: In the seawater surface layer, both turbidity and SPM concentration measurements showed temporal differences only for the dumping sites of Gdańsk and Darłowo. For the bottom layer, a statistically significant temporal differences for the SPM concentrations appeared only for the Gdynia dumping site. However, for this layer, temporal differences in turbidity appeared for all places except DCT.

### 4.2. Concentrations of Nutrients in in the Bottom Layer

Nutrients are an essential element in the ecosystem, and their lack inhibits plant growth and development. However, excessive or unbalanced concentrations of any of the basic elements or nutrients disturb the balance of the ecosystem and lead to eutrophication [15]. The increase in nutrients in waters raises the SPM levels and, consequently, water turbidity.

We applied statistical analyses, including cluster analysis, in order to characterize the biochemical and optical properties of seawater and to find the similarities between the investigated dumping sites in different seasons. For this purpose, we calculated the arithmetic mean of selected parameters that present the general characteristics of the tested areas, and, in a further analysis, we applied “tree structure” and created a dendrogram of cluster analysis.

**Figure 8:**
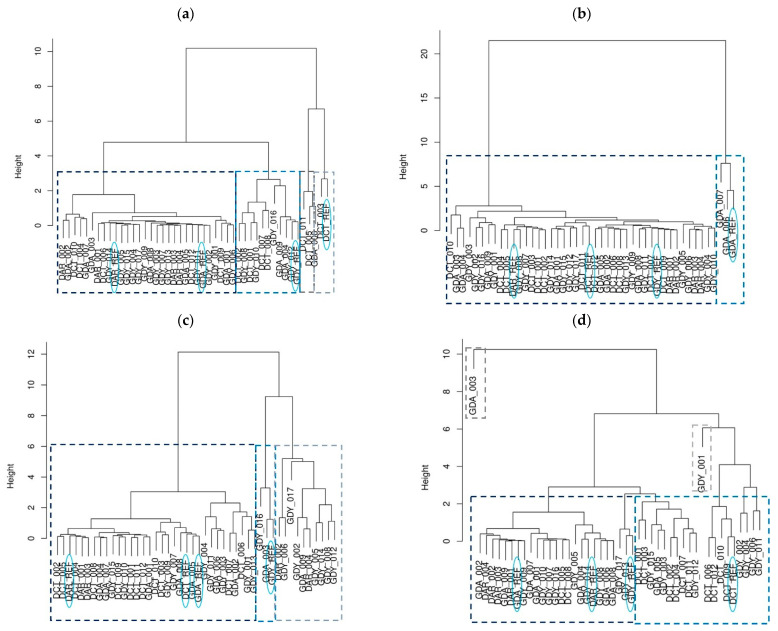
Hierarchical cluster analysis of the sampling sites of the sea dumping sites using biochemical data: (**a**) spring, (**b**) summer, (**c**) autumn, and (**d**) winter.

The results of these measurements were used to group the sea dumping site points with similar properties of nutrients, near-bottom turbidity, and near-bottom SPM. They are presented in Figure 8 as hierarchical clustering dendrograms for each season. In the dendrogram, each leaf corresponds to one offshore dumping station, and the height describes the similarity between individual sea dumping sites. The *y*-axis is a measure of closeness of either individual data points or clusters. We decided to distinguish from two (summer) to four (spring and winter) clustering groups to show the similarities in the concentrations of nutrients, near-bottom turbidity, and near-bottom SPM between the points. One can see that, in each season, the distances between the sea dumping site points are relatively small. Several stepgroups have been recognized in each season. We observe the shortest distances between the clusters in the spring and winter, while the greatest distances are observed in the summer.

Four groups have been recognized in the spring season: two more numerous and two less numerous, composed mostly of DCT points (Figure 8a).

The most numerous group comprises clusters indicated by a dark blue frame and reference points from the Darłowo and Gdańsk sites. The group indicated by a light blue frame includes a reference point of the Gdynia site. The reference point of the DCT site belongs to the second least numerous group (light grey frame). The greatest distances between points are observed in the light blue group. Clusters formed during the summer (Figure 8b) fell into two fairly distinct groups: the first group was small (light blue frame), composed of three GDA points, and it included a reference point of the Gdańsk site. The reference points of the remaining dumping sites, DCT, Darłowo, and Gdynia, belonged to the second group (dark blue frame). The remaining points of the sea dumping sites were assigned to the second group. The distances between the points in both of the clusters were comparable.

For the autumn (Figure 8c), the cluster analysis identified three groups of dumping site points. The smallest group (light blue frame) included only three points: two from the Gdynia dumpsite (including the reference point) and one from the Gdańsk dumpsite. The distances between them were relatively small. The second of the smallest groups (light grey frame) included almost all points from the Gdynia dumpsite (only two points were from the Darłowo dumpsite). The biggest difference was in the distances between GDY_016 or GDY_017 and other points. The reference points of the remaining dumping sites, DCT, Darłowo, and Gdańsk, belonged to the most numerous group (dark blue frame). In this group, the distances between the dumpsite points were comparable.

In the fourth season, the winter (Figure 8d), the cluster analysis indicated seven groups of dumping site points. Two separate groups (in dark blue frames) consisted only of one point of the Gdańsk—GDA_003—and one of the Gdynia—GDY_001—dumping sites, and had distinct dispersal distances. The remaining groups consisted of points with shorter distances between them. Moreover, two less numerous groups in light and dark violet frames were made up only of Gdynia points. Most of the DCT points formed a separate cluster (dark grey frame) with reference points of the DCT and one point of the Gdynia site, GDY_012. Two more numerous groups contain other reference points of dumping sites: Gdańsk, Darłowo and DCT.

The greatest variability in nutrient concentrations, near-bottom turbidity, and near-bottom SPM concentrations was observed in the spring. Additionally, we noticed that at this time of the year, six points from the DCT dumping site (including the reference point) and point GDA_006 formed the two smallest groups (light and dark grey). This is due to their markedly higher percentages of P-tot and P-PO_4_^−3^. We received two groups for the summer and three for the autumn. In the summer, the light blue frame group consisted of only a few points from the Gdańsk dumping site (including the reference point), and they were characterized by high concentrations of all of the nutrients. The second and most abundant group (in the dark blue frame) consisted of points with low concentrations of nitrates and nitrites, combined with the average concentrations of phosphates. This group also included three reference points from the Darłowo, DCT, and Gdynia dumpsites. The near-bottom turbidity and near-bottom SPM concentrations were comparable in all of the summer groups. In the autumn, the dark blue cluster included the dumping site points that were characterized by slightly higher concentrations of biogenic substances (especially concentrations of N-NO_3_^−^) than points from the other clusters. The least numerous light blue cluster included points only from the Gdynia (as well as the reference point) and Gdańsk dumping sites, and was characterized by slightly higher concentrations of nutrients, near-bottom turbidity, and near-bottom SPM concentrations. The light grey cluster included points mostly from the Gdynia dumping site and was characterized by slightly higher values of near-bottom nutrients and near-bottom SPM concentrations. Figure 5d shows that, in the winter, points from all of the dumping sites were divided into two large groups (dark and light blue frames), except for two points from Gdynia and Gdańsk (dark and light grey frames). Interestingly, in the dark blue group, there were all of the points from the Darłowo dumping site (including the reference point) and almost all of the points from the Gdańsk dumping site (except for GDA_005). This group also included reference points from the Gdynia and Gdańsk dumpsites. On the other hand, in the light blue group there were almost all of the points from the DCT dumping site (except for DCT_005 and DCT_008) (including the reference point), and seven points from the Gdynia dumping site. The concentrations of nutrients were rather high and comparable in these two groups, while values of near-bottom T and near-bottom SPM concentrations were rather small. In Figure 5d, we also see two points from the Gdynia and Gdańsk dumping sites (dark and light frames) that are very different from the others. These points are characterized by high values of near-bottom turbidity and near-bottom SPM concentrations (GDY_001: T = 10.62 FTU and SPM = 8.3 mg∙dm^−^^3^; GDA_003: T = 4.27 FTU and SPM = 12.5 mg∙dm^−3^).

In the spring, the highest concentrations of N-tot were for the DCT_003 station, and amounted to 0.28 mg∙dm^−3^; the highest concentrations of N-NO_3_^−^ were for the DCT_010 station, and amounted to 0.059 mg∙dm^−3^; the highest concentrations of N-NH_4_^+^ were for the GDY_001 station, and amounted to 0.07 mg∙dm^−3^; the highest concentrations of P-tot were for the DCT_010 station, and amounted to 0.175 mg∙dm^−3^; and the highest concentrations of P-PO_4_^−3^ were for the DCT_010 station, and amounted to 0.171 mg∙dm^−3^. In the summer, the highest concentrations of N-tot were for the DCT_007 station, and amounted to 0.47 mg∙dm^−3^; the highest concentrations of N-NO_3_^−^ were for the GDY_007 station, and amounted to 0.087 mg∙dm^−3^; the highest concentrations of N-NH_4_^+^ were for the GDY_017 station, and amounted to 0.082 mg∙dm^−3^; the highest P-tot concentrations were for the GDY_011 station, and amounted to 0.136 mg∙dm^−3^; and the highest P-PO_4_^−3^ concentrations were for the GDY_011 station, and amounted to 0.127 mg∙dm^−3^. In the autumn, the highest concentrations of N-tot were for the DAR_002 station, and amounted to 0.23 mg∙dm^−3^; the highest concentrations of N-NO_3_^−^ were for the GDY_REF station, and amounted to 0.084 mg∙dm^−3^; the highest concentrations of N-NH_4_^+^ were for the GDY_010 station, and amounted to 0.031 mg∙dm^−3^; the highest P-tot concentrations were for the DCT_010 station, and amounted to 0.082 mg∙dm^−3^; and the highest P-PO_4_^−3^ concentrations were for the DCT_010 station, and amounted to 0.073 mg∙dm^−3^. In the winter, the highest concentrations of N-tot were for the DAR_004 and DAR_REF stations, and amounted to 0.20 mg∙dm^−3^; the highest concentrations of N-NO_3_^−^ were for the DAR_002 station, and amounted to 0.175 mg∙dm^−3^; the highest concentrations of N-NH_4_^+^ were for the DCT_004 station, and amounted to 0.01 mg∙dm^−3^; the highest concentrations of P-tot were for the DCT_012 station, and amounted to 0.128 mg∙dm^−3^; and the highest concentrations of P-PO_4_^−3^ were for the DCT_003 station, and amounted to 0.124 mg∙dm^−3^.

We compared the obtained results of the average concentrations of nutrients in the water collected from the Darłowo dumpsite and the reference point with the limit values for surface water quality classes specified in Annex 24 to the Regulation of the Minister of the Environment [43]. It turned out that the limit values for N-tot and P-PO_4_^−3^ were exceeded at all four of the dumpsites. Moreover, the mineral nitrogen limits were exceeded at the Darłowo dumpsite, and the P-tot levels were exceeded at the dumpsites located in the Gdańsk Bay.

### 4.3. Limitations of Our Research and Recommendations for the Future Research

This particular study has some limitations due to the sample size: survey campaigns were carried out only once in each season. To confirm our results and observations, it is recommended to repeat the research in the following years. Moreover, the work does not take into account the parameters that describe the dynamics of the studied areas, such as waves or wind speed and direction. However, our results are simply an introduction to the planning of further measurements of turbidity, which has never been studied in this area in such detail and scope.

In the case of coastal zone waters and internal seas, such as the Baltic Sea, the correlations between optical properties and the concentrations of SPM are much weaker than in open ocean waters, and they are often purely local (which is particularly visible in the areas of dumpsites). These sea areas are characterized by high biological productivity and are important, although still insufficiently known, elements of the environment.

The basic problem in developing algorithms for these waters is the complexity of their optical properties [17]. Coastal and bay waters are not only optically but also chemically more complex than open sea areas. They are highly variable in their physicochemical properties and contain much more molecular and dissolved matter than open ocean waters do. In such sea areas, rivers bring large infusions of freshwater, which introduce a lot of different dopants, such as particulate organic matter (POM) and particulate inorganic matter (PIM), as well as nutrients. Nutrients have a strong impact on phytoplankton biomass, which in turn affects the amount and type of SPM in seawater. Therefore, single-parameter relationships presented in the form of linear equations (see Equation (1)), given by coefficients placed in Figure 6 and Figure 7, are not sufficient to describe the turbidity of the considered sea areas of the Southern Baltic. For this reason, the dependencies obtained in the first stage of our work should be subject to subsequent statistical analyses, during which additional parameters could be introduced, e.g., concentrations of POM and PIM. We have not measured these parameters so far, but we intend to do so in the future. We believe that this will make our analyses more effective.

Since the values of optical properties in natural waters are primarily the result of suspended matter and substances dissolved in the water, they can differ significantly depending on the percentage of each of these optically active factors. In the sea areas where dissolved organic substances dominate (e.g., bay waters and the dumpsites of Gdynia, Gdańsk, and DCT), much better results are likely to be obtained by approximating the optical properties based on the concentration of dissolved or suspended organic matter. On the other hand, in the case of waters with a predominance of mineral particles (shallow open coastal waters, e.g., the Darłowo dumpsite), much better results are obtained by approximating the optical properties by using the PIM concentration.

However, both in the case of the dumpsites located within the Gdańsk Bay and the coastal waters of the Southern Baltic, the multiparameter dependencies of optical coefficients on the concentrations of water components will be more effective than single-parameter dependencies. Therefore, since within coastal and bay waters the values of optical properties may vary within several orders of magnitude, it is most reasonable to establish for each of these reservoirs, first of all, individual and seasonal links between optical properties and the concentrations of individual components identified in their waters.

## 5. Conclusions

This study marks a first attempt in the characterization of turbidity, measured in FTU, in four dumping sites located in Polish coastal waters, using empirical data collected by an STD/CTD probe. Our research is the first such detailed study on turbidity in the dumping areas of the Southern Baltic. In addition, SPM contents in the surface and bottom water layers as well as the concentrations of nutrients in bottom waters were investigated. The research was carried out in the course of four measurement campaigns (spring, summer, autumn, and winter), which made it possible to investigate the temporal variability in the parameters examined.

The results obtained differed between individual dumpsites, but we noticed certain trends: lower turbidity values recorded in the spring and winter, and higher ones recorded in the summer and autumn in the dumpsites of the Gdańsk Bay. At the dumping site of Darłowo, located in a high-water-dynamics area, which favors the mixing of water in vertical planes and exchange with waters of the Southern Baltic, we recorded higher values in the spring and winter as well as lower values in the summer and autumn. High values of nutrients were recorded at all of these dumpsites. In 62% of the cases, the mean turbidity measured in the area of a dumpsite was higher than at its reference point. It is not a rule that within a dumpsite we observe a higher level of turbidity than in adjacent waters or at the reference points. At this stage of the research, however, it cannot be stated unequivocally that the situation identified is influenced solely by the dumpsites or rather the specific features of the areas in which they are located.

Optical methods are now widely used to monitor the world’s oceans via satellites. Algorithms for estimating various biotic and abiotic parameters in coastal and bay waters are much more complex than those applied for open sea waters, and they can be used only locally. Because areas of dumpsites are often strategically important, it is crucial to find the closest possible local relationships between the components of seawater and its optical properties. To improve accuracy in estimating the concentrations of optically significant components in coastal and bay waters using remote sensing methods, it is necessary, first of all, to know the biochemical and optical properties of these components well. Seawater constituents, such as nutrients, determine the phytoplankton, POM, and PIM contents, and these, in turn, affect the optical properties.

In the future, our analyses, correlating turbidity and SPM concentrations, could form the basis for local remote sensing algorithms, allowing for the estimation of the concentration values of SPM in water based on the knowledge of its optical properties, e.g., light attenuation in seawater.

Moreover, the juxtaposition of these four optically complex reservoirs significantly broadened our knowledge of the nature of the processes related to the transmission of light energy in the waters of dumping sites and provided interesting results. In the future, our results may help in the remote sensing monitoring of dumpsite areas as well as bay and coastal areas, as well as constitute a fundamental contribution to the construction of bio-optical algorithms for these sea areas. Thanks to the obtained results, it will be possible to control the ecosystem and the state of water in these areas more precisely and effectively, i.e., their environmental monitoring.

An equally important effect of our research is a very extensive empirical database, which extends the knowledge on temporal changes in the values of the parameters tested, i.e., turbidity, SPM, and nutrients.

## Figures and Tables

**Table 1:** Transects with the name of the place of measurement, the length, and the direction.

Transect	Dumpsite	Measurement Points	Length	Course
AB	Gdynia	GDY_018, GDY_015, GDY_007, GDY_008, GDY_009, GDY_017, and GDY_020	5.0 km	W–E
CD	Gdynia	GDY_021, GDY_014, GDY_008, GDY_001, GDY_016, GDY_019, and GDY_ref	3.6 km	S–N
EF	Gdańsk, DCT	GDA_009, GDA_001, GDA_007, DCT_013, DCT_012, DCT_004, DCT_005, DCT_010, and DCT_015	8.0 km	W–E
GH	Gdańsk	GDA_010, GDA_005, GDA_001, GDA_003, GDA_012, and GDA_ref	4.45 km	NW–SE
IJ	DCT	DCT_016, DCT_011, DCT_007, DCT_002, DCT_009, DCT_014, and DCT_ref	3.9 km	S–N
KL	Darłowo	DAR_008, DAR_007, DAR_003, DAR_001, DAR_002, DAR_005, and DAR_006	2.0 km	SW–NE
MN	Darłowo	DAR_001, DAR_004, DAR_009, and DAR_ref	2.0 km	SE–NW

**Table 2:** Concentration of nutrients for the near-seabed layer averaged for all of the sampling sites of the given dumpsites (before the slash) versus data coming from the reference points (after the slash); “<” if a result is below the lower limit of quantification.

Sea Dumping Site	Season	N-tot (mg∙dm^−3^)	N-NO_3_^−^ (mg∙dm^−3^)	N-NH_4_^+^ (mg∙dm^−3^)	P-tot (mg∙dm^−3^)	P-PO_4_^−3^ (mg∙dm^−3^)
Gdynia	Spring	0.15/0.14	<0.003/<0.003	0.017/< 0.003	0.028/0.026	0.032/0.047
	Summer	0.33/0.28	0.025/0.032	0.025/0.030	0.032/0.030	0.022/0.020
	Autumn	0.13/0.16	0.040/0.084	0.013/0.017	0.031/0.060	0.024/0.055
	Winter	0.14/0.13	0.052/0.050	<0.003/0.009	0.024/0.030	0.020/0.029
Gdańsk	Spring	0.16/0.14	<0.003/<0.003	0.011/<0.003	0.029/0.027	0.011/0.007
	Summer	0.32/0.29	0.028/0.026	0.027/0.030	0.027/0.024	0.022/0.016
	Autumn	0.12/0.11	0.027/0.019	0.019/0.027	0.015/0.010	0.009/0.010
	Winter	0.14/0.11	0.047/0.045	<0.003/<0.003	0.023/0.017	0.018/0.013
DCT	Spring	0.19/0.23	0.035/< 0.003	0.009/<0.003	0.079/0.091	0.062/0.036
	Summer	0.29/0.21	0.059/0.043	0.025/<0.003	0.075/0.048	0.066/0.042
	Autumn	0.12/0.14	0.049/0.048	0.009/<0.003	0.066/0.069	0.060/0.062
	Winter	0.12/0.14	0.046/0.031	<0.003/<0.003	0.057/0.040	0.054/0.034
Darłowo	Spring	0.18/0.17	<0.003/0.007	<0.003/<0.003	0.026/0.033	0.014/0.021
	Summer	0.34/0.28	<0.003/<0.003	0.008/0.004	0.018/0.018	<0.009/0.016
	Autumn	0.19/0.18	0.058/0.021	0.008/0.015	0.027/0.025	0.018/0.015
	Winter	0.17/0.20	0.126/0.109	<0.003/<0.003	0.024/0.025	0.022/0.019

**Table 3:** Turbidity and SPM concentrations averaged for all of the sampling sites of the given dumpsites and the appropriate layer (before the slash) versus data averaged at the reference points (after the slash).

Sea Dumping Site	Season	Turbidity (Surface Layer) (FTU)	Turbidity (Seabed Layer) (FTU)	Turbidity in the Entire Water Column (FTU)	SPM (Surface Layer) (mg∙dm^−3^)	SPM (Seabed Layer) (mg∙dm^−3^)
Gdynia	Spring	3.74/0.30	1.30/5.68	0.36/1.70	2.72/0.44	2.81/2.00
	Summer	1.40/1.01	1.97/1.09	0.68/0.55	1.87/1.00	3.07/1.00
	Autumn	3.37/13.21	7.29/16.60	1.59/1.13	5.19/6.66	5.64/8.10
	Winter	2.28/0.24	2.56/0.26	0.55/0.25	2.16/0.38	2.72/4.20
Gdańsk	Spring	3.53/7.73	2.11/0.20	0.28/0.39	2.89/1.78	2.11/0.38
	Summer	2.75/1.67	8.66/0.58	1.40/0.55	3.49/2.34	2.52/0.56
	Autumn	0.91/0.72	4.90/0.17	1.09/0.27	0.87/1.99	1.32/0.38
	Winter	0.30/1.84	0.30/0.37	0.26/0.28	0.37/0.46	1.19/1.99
DCT	Spring	1.29/2.00	2.80/7.23	0.25/0.16	2.00/1.00	3.96/12.56
	Summer	1.25/0.98	1.27/1.37	0.38/0.33	2.41/2.30	2.19/1.65
	Autumn	3.65/0.37	3.13/1.55	0.37/0.37	2.07/0.48	2.17/1.55
	Winter	2.61/0.25	2.88/2.84	0.34/0.77	2.14/0.38	4.80/<2.84
Darłowo	Spring	3.85/4.79	0.29/0.24	0.40/0.41	4.15/1.24	0.99/0.24
	Summer	0.44/0.67	0.24/0.09	0.25/0.11	0.55/1.10	0.63/0.09
	Autumn	5.35/4.22	0.60/0.34	0.30/0.23	3.28/2.48	1.00/0.34
	Winter	1.78/9.90	2.69/0.33	0.42/0.55	4.88/8.50	3.83/0.38

**Table 4:** Percentage of measurement points where the results of turbidity and SPM concentration exceeded those obtained for the reference points.

Sea Dumping Site/Number of Sampling Points	Season	Turbidity in Surface Layer (%)	Turbidity in Bottom Layer (%)	SPM in Surface Layer (%)	SPM in Bottom Layer (%)
Gdynia 17	Spring	88	12	82	6
	Summer	35	88	47	65
	Autumn	0	6	0	6
	Winter	100	65	88	24
Gdańsk 9	Spring	22	89	56	100
	Summer	78	100	67	100
	Autumn	33	100	0	100
	Winter	0	11	22	11
DCT 12	Spring	25	8	75	8
	Summer	83	25	58	58
	Autumn	92	42	92	50
	Winter	92	50	92	75
Darłowo 4	Spring	25	25	75	25
	Summer	0	100	0	100
	Autumn	50	75	50	75
	Winter	0	100	0	100

**Table 5:** Shapiro–Wilk test and *t*-test results. W values higher than the critical value mean that the distribution of the SPM and turbidity is normal. For these cases, the *t*-test is applied. The results of a *t*-test test the hypothesis of whether there are significant differences between the values at the reference points and the other measurement points. Values lower than the critical value, t(α, n − 1), mean no significant differences.

Sea Dumping Site/Number of Sampling Points	Critical Values $W\left(\alpha ,n\right)$ $t\left(\alpha ,n-1\right)$	Season	W_SPM Surface_ t_SPM Surface_	W_T Surface_ t_T Surface_	W_SPM Bottom_ t_SPM Bottom_	W_SPM Bottom_ t_SPM Bottom_
Gdynia 17	0.892 2.120	Spring	0.699	0.660	0.746	0.581
			-	-	-	-
		Summer	0.813	0.611	0.861	0.957
			-	-	-	0.981
		Autumn	0.751	0.850	0.653	0.903
			-	-	-	−1.560
		Winter	0.939	0.774	0.90	0.76
			1.306	-	−0.726	-
Gdańsk 9	0.829 2.306	Spring	0.821	0.716	0.709	0.749
			-	-	-	-
		Summer	0.948	0.858	0.770	0.723
			0.606	0.825	-	-
		Autumn	0.824	0.588	0.936	0.757
			-	-	1.259	-
		Winter	0.944	0.633	0.845	0.46
			−0.611	-	−1.571	-
DCT 12	0.859 2.201	Spring	0.880	0.875	0.768	0.859
			0.691	−0.632	-	−1.697
		Summer	0.882	0.901	0.879	0.888
			0.184	0.876	0.452	−0.355
		Autumn	0.855	0.674	0.685	0.573
			-	-	-	-
		Winter	0.761	0.717	0.92	0.85
			-	-	0.564	-
Darłowo 4	0.748 3.182	Spring	0.800	0.991	0.668	0.640
			0.598	−0.305	-	-
		Summer	0.964	0.989	0.938	0.837
			−2.194	−1.738	1.624	1.674
		Autumn	0.820	0.855	0.844	0.781
			0.396	0.468	0.620	0.564
		Winter	0.919	0.887	0.92	0.78
			−1.958	−6.990	5.775	4.478

**Table 6:** Kruskal–Wallis test results. For H values higher than the critical value, we rejected the null hypothesis that there were no statistical differences between the measurements at different times of the year. For lower values, there was no reason to reject this hypothesis.

Sea Dumping Site	Critical Value of the Kruskal–Wallis Test	H_SPM Surface_	H_T Surface_	H_SPM Bottom_	H_T Bottom_
Gdynia	7.815	2.213	2.801	11.260	24.305
Gdańsk		18.631	13.829	1.375	17.867
DCT		2.707	0.962	3.335	0.961
Darłowo		8.140	8.670	8.625	10.478

## Data Availability

The data that support the findings of this study are available from the first author upon justified request.
